# Banti's Disease

**Published:** 1902-09-06

**Authors:** 


### BANTI'S DISEASE.

It is now twenty years since Professor Banti of Florence described the condition which has since been known as Banti's disease. Under the generic name of splenic anæmia doubtless a considerable number of separate diseases are included. Although many writers use the terms "Banti's disease," "splenic anæmia," "splenic cachexia," "splenic pseudo-leukæmia," "lymphadenoma splenica," and "splenomegalia primitiva," as synonymous, Dr. James Barr[1] holds, and probably very correctly holds, that under these various designations many varieties of disease have been included, the conditions common to all of which have been a large spleen with more or less well-marked anæmia. According to Banti's description of the malady to which his name has been attached, the clinical features were anæmia with enlargement of the spleen and secondary cirrhosis of the liver with ascites; frequent hæmorrhages, such as epistaxis, bleeding of the gums, hæmatemesis, and melæna. Pathologically he recognised fundamental and striking changes in the sympathetic nervous system, consisting of lymphoid infiltration of the stroma of the ganglia, fatty and pigment degeneration of the ganglion cells, and a degeneration of the nerve fibres springing from the ganglia; these changes being found not only in the semilunar ganglia and solar plexus of the abdomen, but also in the ganglia of the neck. The changes in the chyle and hæmopoietic organs (as the stomach, bowels, spleen, and liver), had a great influence on nutrition, so that the cause of the anæmia which was so striking a symptom in the cases was very apparent. He regarded the tumefaction or engorgement of the spleen and liver as the primary condition, and the fibrosis of these organs which followed as a secondary consequence. Dr. Barr relates three cases of this disorder, and in summing up says that his view of Banti's disease is that it is probably due to a vaso-motor paresis of the splanchnic area, either in whole or in part, and that this paresis arises from disease of the visceral sympathetic ganglia. As a consequence of this there is great engorgement of the abdominal viscera, especially of the spleen and liver, increased hæmolysis, with consequent oligochromæmia and oligocythæmia. The increased blood supply to those organs eventually leads to fibrosis and lessened function. The paresis also leads to retention of blood in the portal area with lessened supply to the rest of the body and general impairment of nutrition. The peritoneal effusion would seem to be due to vascularity rather than to portal obstruction.

[1] Lancet, Aug. 23.

